# Increased Recognition of Human Anaplasmosis, Ontario, Canada, 2021

**DOI:** 10.3201/eid3104.231435

**Published:** 2025-04

**Authors:** Cathy Dai, David Good, Andreea Slatculescu, Manisha A. Kulkarni, T. Hugh Guan, Evan Wilson, Siddhartha Srivastava

**Affiliations:** Queen’s University, Kingston, Ontario, Canada (C. Dai, D. Good, E. Wilson, S. Srivastava); Kingston Health Sciences Center, Kingston (C. Dai, D. Good, E. Wilson, S. Srivastava); University of Ottawa School of Epidemiology and Public Health, Ottawa, Ontario (A. Slatculescu, M.A. Kulkarni); Kingston, Frontenac, and Lennox & Addington Public Health, Kingston, Ontario (T.H. Guan)

**Keywords:** Anaplasmosis, *Anaplasma phagocytophilium*, human granulocytic anaplasmosis, tickborne diseases, vector-borne infections, bacteria, Canada

## Abstract

Human granulocytic anaplasmosis is a tickborne infection characterized by fever, thrombocytopenia, leukopenia, transaminitis, or a combination of those. Treatment must be prompt and appropriately targeted to prevent clinical decompensation. We discuss an unusual cluster of 16 probable cases in Ontario, Canada, during June–August 2021.

Human granulocytic anaplasmosis (HGA) is a tickborne infection caused by the intracellular, gram-negative bacteria *Anaplasma phagocytophilium *(*1*). The infection is transmitted to people via bites from infected blacklegged ticks (*Ixodes scapularis*) (*1*). HGA has been historically limited to the northeastern and upper midwestern portions of the United States (*2*). However, factors such as climate change (*3*,*4*) and the spread of ticks via migratory birds from the United States to Canada (*5*) have contributed to the northward spread of the range of blacklegged tick populations at an estimated rate of 33–55 km/year (*4*). Reports of anaplasmosis in Canada emerged in 2009 (*6*), and the first locally acquired case was reported in eastern Ontario in 2018 (*7*). We describe a cluster of 16 probable anaplasmosis cases requiring admission to an academic hospital in eastern Ontario, Canada, during June–August 2021.

At the time of our study, anaplasmosis was not a reportable disease of public health concern in Ontario. It became reportable in 2023. We identified cases using data abstraction from electronic health records. We flagged adults >18 years of age who visited a tertiary care hospital in eastern Ontario and whose records showed hematopathologist-reported inclusions on peripheral blood smears. We performed manual abstraction and verification and collected demographic data, clinical notes, and diagnosis and treatment data for descriptive analysis. We performed geographic spatial visualization using the forward sortation area, and we overlaid those data with tick-dragging data from public health units (Appendix). We obtained ethics approval through the Queen’s University Research Ethics board.

We identified 16 probable cases of HGA infection as defined by US Centers for Disease Control and Prevention criteria (*8*). Of note, none met the Centers’ case definition for confirmed HGA infection because of a lack of follow-up serology or PCR testing. Fourteen (87.5%) cases met supportive laboratory criteria, with morulae seen in neutrophils on peripheral blood smear (Figure). Two (12.5%) cases had a single indirect fluorescent antibody IgG titer of >1:64. None of the cases involved PCR testing. The mean age of case-patients was 69 (range 32–91; median 70) years, and the mean Charlson Comorbidity Index was 3.3. Seven case-patients were female, and 9 were male.

**Figure:**
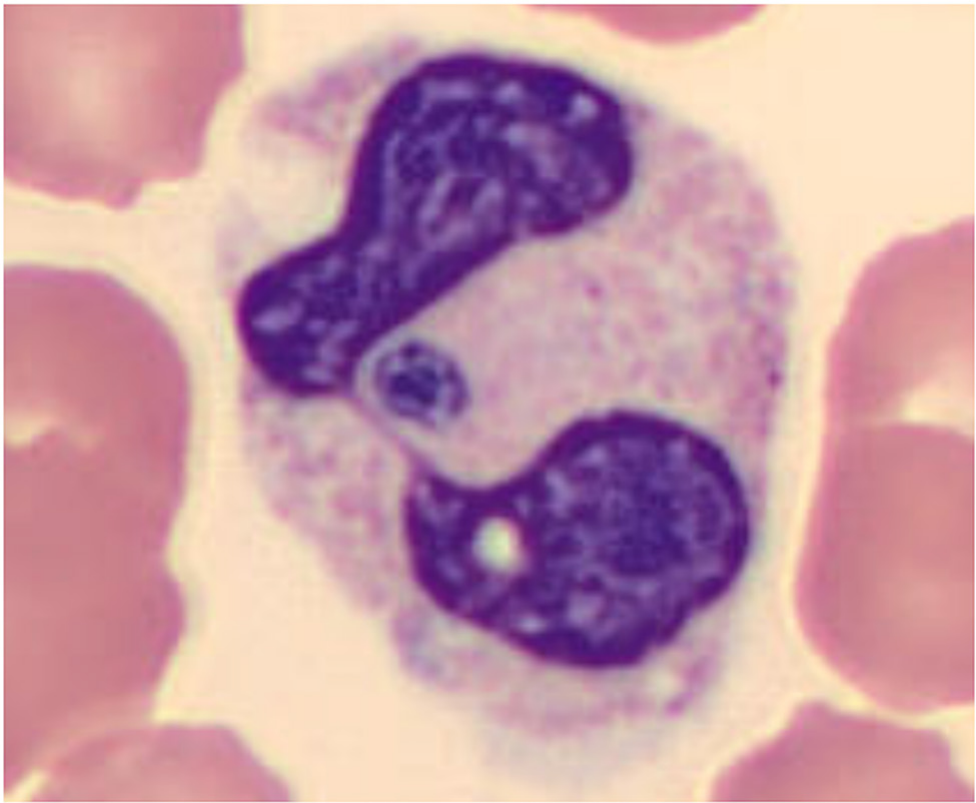
Peripheral blood smear from a sample studied as part of an investigation into increased recognition of human anaplasmosis, Ontario, Canada, 2021. Morulae (deep purple inclusions) can be visualized within the neutrophil. Wright’s stain, original magnification ×100.

All case-patients were febrile and had laboratory abnormalities, most commonly transaminitis (85%) or thrombocytopenia (76%). Ten (62.5%) case-patients required hospital admission, and median length-of-stay was 5 (range 3–13) days. Clinical deterioration in patients corresponded with delays in appropriate treatment.

Two (12.5%) case-patients required intensive care unit (ICU) admission. Both were elderly (73 and 70 years of age), and both had a Charlson Comorbidity Index of 4, slightly above the 3.3 mean. Both patients entered the hospital with initially stable, undifferentiated febrile illness. They decompensated over 3–4 days, requiring transfer to the ICU without a clear diagnosis of HGA infection. One patient developed severe hypoxic respiratory failure and the other progressed to septic shock, requiring vasopressor support. Both patients encountered associated complications, including new atrial fibrillation, acute renal injury, and hyponatremia. Administration of doxycycline resulted in clinical improvement within 48 hours, and both patients were able to transfer out of the ICU. We observed similar prompt improvement across all cases in which patients received appropriate treatment. 

HGA is typically mild and self-limiting, but patients can develop life-threatening complications, particularly if they are elderly, have comorbidities, or if they experience delays in diagnosis and treatment (*2*). The number of patients with serious complications requiring hospitalization and ICU admission seen here likely reflects delayed clinician recognition of HGA. This series of 16 probable HGA cases identified in 1 hospital over 3 months raises concern that infection rates are increasing locally. Because of a lack of PCR confirmation or follow-up serology in the cases we reviewed, a limitation of this study was that none of the probable cases could be considered epidemiologically confirmed. Research suggests, however, that tickborne infections like HGA will continue to become more prevalent throughout Canada (*3*,*4*,*6*). High incidences of blacklegged ticks and Lyme disease are known in southeastern Ontario (*9*). 

In 2023, the first year that HGA was reportable in Ontario, researchers identified 40 cases of anaplasmosis, with 17 cases confirmed (*10*). Those data likely emerged consequentially with increased rates of PCR and serology testing and increasing local HCP awareness of HGA resulting from public health involvement. Our study demonstrates the potential severity of HGA infections in cases where diagnosis and appropriate treatment is delayed and illustrates how continuing advancements in education of HCPs could help in increasing recognition of this emerging infectious disease in Canada. 

In conclusion, healthcare providers (HCPs) should consider HGA when treating patients with fever, thrombocytopenia, transaminitis, or leukopenia during spring, summer, and fall. When encountering potential cases, HCPs should treat promptly with doxycycline rather than await confirmatory testing results. Improved awareness regarding the most appropriate confirmatory testing for HGA would help HCPs in establishing a diagnosis. In treating patients in the acute phase of illness, HCPs should request PCR testing, rather than serology. 

Appendix Additional information for increased recognition of human anaplasmosis, Ontario, Canada, 2021.
