# Supplementary material for: Increased Recognition of Human Anaplasmosis, Ontario, Canada, 2021
Source: Emerg Infect Dis. 2025 Apr;31(4):849–51. doi: 10.3201/eid3104.231435 (PMC11950283; doi:10.3201/eid3104.231435)
Supplement: Appendix — Additional information for increased recognition of human anaplasmosis, Ontario, Canada, 2021. [file 23-1435-Techapp-s1.pdf]

Article DOI: http://doi.org/10.3201/eid3104.231435

EID cannot ensure accessibility for supplementary materials supplied by authors. Readers who have difficulty accessing supplementary content should contact the authors for assistance.

Increased Recognition of Human Anaplasmosis, Ontario, Canada, 2021

Appendix

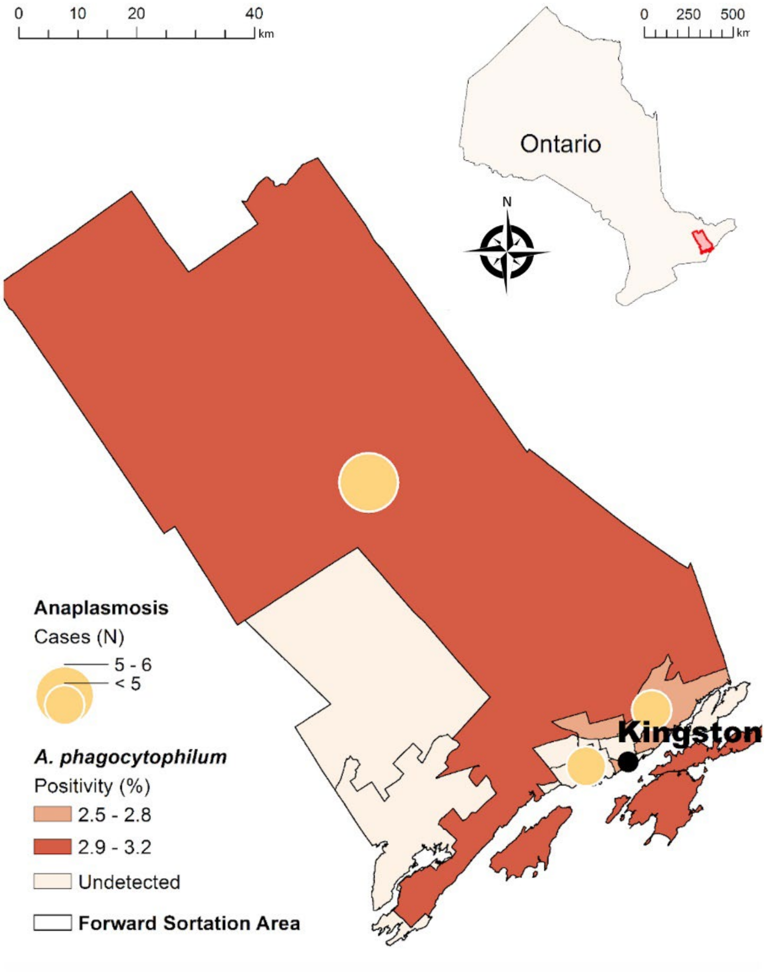

Appendix Figure. Anaplasmosis case numbers overlaid on *Anaplasma phagocytophilum* positivity in ticks by forward sortation area in a study of increased recognition of human anaplasmosis, Ontario, Canada, 2021.
